# Reading text works better than watching videos to improve acuity in a simulation of artificial vision

**DOI:** 10.1038/s41598-022-10719-6

**Published:** 2022-07-28

**Authors:** Katerina Eleonora K. Rassia, Konstantinos Moutoussis, John S. Pezaris

**Affiliations:** 1 Cognitive Science Laboratory, Department of History and Philosophy of Science, National and Kapodistrian University of Athens, Athens, Greece; 2 Department of Neurosurgery, Massachusetts General Hospital, Boston, MA USA; 3 Department of Neurosurgery, Harvard Medical School, Boston, MA USA

**Keywords:** Perception, Reading, Sensory processing, Visual system, Translational research

## Abstract

Simulated artificial vision is used in visual prosthesis design to answer questions about device usability. We previously reported a striking increase in equivalent visual acuity with daily use of a simulation of artificial vision in an active task, reading sentences, that required high levels of subject engagement, but passive activities are more likely to dominate post-implant experience. Here, we investigated the longitudinal effects of a passive task, watching videos. Eight subjects used a simulation of a thalamic visual prosthesis with 1000 phosphenes to watch 23 episodes of classic American television in daily, 25-min sessions, for a period of 1 month with interspersed reading tests that quantified reading accuracy and reading speed. For reading accuracy, we found similar dynamics to the early part of the learning process in our previous report, here leading to an improvement in visual acuity of 0.15 ± 0.05 logMAR. For reading speed, however, no change was apparent by the end of training. We found that single reading sessions drove about twice the improvement in acuity of single video sessions despite being only half as long. We conclude that while passive viewing tasks may prove useful for post-implant rehabilitation, active tasks are likely to be preferable.

## Introduction

Contemporary visual prostheses provide only a crude approximation to normal vision, thus post-implant therapies play an important role in an over-all treatment plan. The ideal rehabilitation strategy for patients receiving visual prostheses remains an open question: what sort of activities would best assist the recipients to adapt to their new visual modality? To help answer such questions, we have previously studied improvements in visual acuity through near-daily use of an active, reading task with normal, sighted subjects viewing a simulation of artificial vision^1^. Here, we extend that work to study the effects of a passive, video viewing task that does not require the same levels of engagement and concentration. We postulate that at-home passive tasks are likely to occupy a larger fraction of daily living than clinic-based active tasks, so it may prove advantageous to incorporate passive tasks into a comprehensive therapeutic strategy.

### Predicting influence of passive tasks versus active tasks

The idea that active engagement is required to drive Visual Perceptual Learning (VPL) is rooted in the intuitive notion that unbridled visual plasticity needs to be tempered to prevent the constant barrage of visual input leading to an undesirable outcome. The tempering force that would modulate VPL is canonically thought to be the conscious effort of attention (see review by Sasaki and colleagues^2^), but many investigations have now shown that even without attention, VPL remains possible^3–8^. Similar modulatory roles are thought to be played by motor action that reinforces accompanying visual perception^9^ and feedback as to the correctness of responses^10^, both factors serving to amplify VPL. The effect is seen for feedback even when that feedback is only internal such as successful recognition of an object^11,12^. Underscoring the importance of modulation of VPL are experiments that bridge the gap between engagement and passivity by recording active experience of one group of subjects in a visually-based virtual reality exploration task and then playing those recordings to a second group of subjects to create passive experiences^13–16^. This comparison using otherwise identical stimuli showed that the effects of active versus passive engagement, such as the ability to identify and remember visual targets or features, are best characterized as graded rather than absolute. We are left, therefore, with the expectation that a passive task lacking feedback would drive VPL more slowly than an active task that included it.

### Quantitative assessment of performance

To predict the potential for improving device utility through VPL, and to suggest rehabilitation training profiles, the field has used simulations of artificial vision with normal-sighted subjects performing psychophysical tasks. These tasks typically employ simple quantifiable behaviours (visual recognition, reading, visuo-motor interaction) in virtual reality setups that simulate the way vision would appear through a prosthesis to measure some aspect of visual perception as a model for blind individuals with implanted devices. Like other researchers, we use visual acuity as the primary performance metric as it is widely understood and recognized in scientific and clinical settings, as well as among the lay population.

Adaptation to artificial vision has been directly studied, or observed as a secondary finding, in many previous studies using active tasks. Experimental paradigms that have been used include visually-guided mobility and navigation^17–20^, object and face recognition^21,22^, letter recognition^23–26^, reading^1,27–31^ or combinations of the above^32,33^. Reading tasks^34^ in particular have proven to be a robust means to assess visual acuity in our laboratory^1,31,35^, and we continue their use here to measure the effects of training.

### Previous reports of learning effects in simulations of artificial vision

Through a line of inquiry using simulated artificial vision, our laboratory has observed instances of VPL in both human^1,25,31^ and animal models^26^. Our most extensive study thus far has been a longitudinal experiment^1^ that assessed both accuracy and speed of reading performance through a simulation of artificial vision^31,36^. Subjects read 40 novel sentences per day conforming to the MNREAD criteria^34^ for 40 sessions over approximately 8 weeks. This training resulted in a general doubling of equivalent visual acuity across the population, with substantial improvements in both reading accuracy and reading speed. Prior to that work, hints of learning effects had been found over a brief period of exposure with humans^25^ and more substantial effects over longer periods with non-human primates^26^.

### Learning effects with reading tasks from other laboratories

Other groups have found that reading under simulations of artificial vision improves with practice for a wide range of conditions. In an early study, Hayes and colleagues found that multiple sessions lead to a twofold increase in reading speed with a hand-manipulated camera^32^. Sommerhalder and colleagues observed impressive increases in reading accuracy and reduced response times in reading 4-letter words, after 1 month of daily training with a retinally-stabilized eccentric field^28^. The same group expanded their study to full-page reading and reported improvements with daily training that asymptoted after 2 months^29^. Dagnelie and colleagues also found that reading short paragraphs of text through a pixelized display benefits from practice for a variety of difficulty levels^30^. Fu and colleagues reported that a similar brief course of daily practice improved reading performance across difficulty levels^24^. Pérez Fornos and colleagues demonstrated that training of more than 1 month allowed subjects to read at 15° eccentricity with the same accuracy as with central reading, but interestingly also with improved performance on other visuo-motor tasks^18^.

### Examples of VPL in non-reading tasks from other groups

In addition to reports using reading tasks, examples of VPL for a wide range of non-reading tasks have been published in studies that simulated artificial vision, typically demonstrating measurable effects within a small number of sessions. For instance, Xia and colleagues reported that multi-object recognition improves after 5 days of 2-h sessions^22^, and Chen and colleagues reported the ability to identify Landolt C orientation plateaus between 15 to 20 sessions^23^. Dagnelie and colleagues described decreasing error rates and completion times for object counting and placement tasks using checkers on a checkerboard with 17 or fewer daily 1-h sessions^17^. In a related study, Srivastava and colleagues described quite substantial decreases in completion time for a similar object placement task along with maze explorations^19^. In contrast, van Rheede and colleagues found trends toward improvement for object placement and wayfinding with three sessions that did not obtain significance^33^, and without confidence that they measured improved perception rather an increased familiarity with the practiced tasks^20^. Finally, Thompson and colleagues found no change in accuracy, but a substantial decrease in response time in a single session of nearly 200 trials of a face recognition task^21^.

### Observations of learning effects in clinical trials of artificial vision

A small handful of reports have studied learning in a clinical setting with recipients of implanted retinal visual prostheses. Patients with the Alpha IMS or AMS devices (Retina Implant AG) showed substantial learning effects on a wide range of behavioral tests^37^ that span from generally improved visuomotor abilities or elimination of nystagmus initially preventing successful fixation to visual objects^38^ to recognizing small words and shapes (1.39 logMAR, 2.2 logMAR of visual acuity respectively) following an intensive 5-day training regime, 3 years after implantation^39^. Patients with the Argus II device (Second Sight Medical Products, Inc.) showed improvements in performance over the first weeks of use in a battery of tasks^40^ that tended to wane over extended time^41^, although Castaldi and colleagues showed a positive correlation in performance in a detection task vs time since implant^42^. The Argus II results were likely confounded by the training regimen required for implant recipients^43^.

### Combining a passive activity and an active assessment

Previous work across the field has largely focused on tasks that require concerted effort rather than ones that do not, despite the expectation that passive viewing will dominate real post-implant experience. To address this gap, we investigated VPL while using a simulated visual prosthesis through the passive experience of video viewing. Given the substantial improvements we previously reported with the active task of reading^1^, and reports from the literature showing the skill of seeing through artificial vision to be highly responsive to training, we hypothesized that the passive task of video viewing could be used to drive a similar effect, but that the learning rate might be slower. We repurposed the reading task for its readout of acuity rather than its training value, and interleaved it sporadically with a video viewing task. Although we minimized the time spent with the reading task by reducing its length and presenting it only infrequently, the tests still represented some experiential time, so we expected effects to remain from them.

To compensate for training originating from both video and reading tasks, we designed a stutter-step schedule in which we systematically varied the placement of a handful of reading tests (R) within the larger set of video-viewing sessions (v), so as to create spans within the overall sequence with either three (R, v, v, v, R; abbreviated as RvvvR) or six (RvvvvvvR) video sessions between reading tests. This schedule, carefully syncopated across subjects, allowed us to measure acuity improvements for runs of consecutive video viewing sessions bounded by single reading tests. We used data from the two lengths of video viewing chains, RvvvR and RvvvvvvR, to linearly decode *β*_V_ and *β*_R_, the gain factors driving changes in acuity due to video viewing and reading, respectively (see “Methods”).

## Results

Eight subjects (8 total; 2 male, 6 female), recruited from students at the University of Athens, Greece, who had documented and assessed ability in reading English above that required for the tasks, completed the experiment. Subjects had self-reported normal or corrected-to-normal visual acuity, without any major visual defect. A ninth subject was disqualified due to gaze tracking issues, and their data are not reported here. Each subject came to the laboratory for a total of 23 sessions; the first session included a Snellen acuity assessment; subsequent sessions included a reading test through our simulation of artificial vision, and/or watching an episode of a television program shown through the same artificial vision simulation.

### Reading accuracy

**Figure 1:**
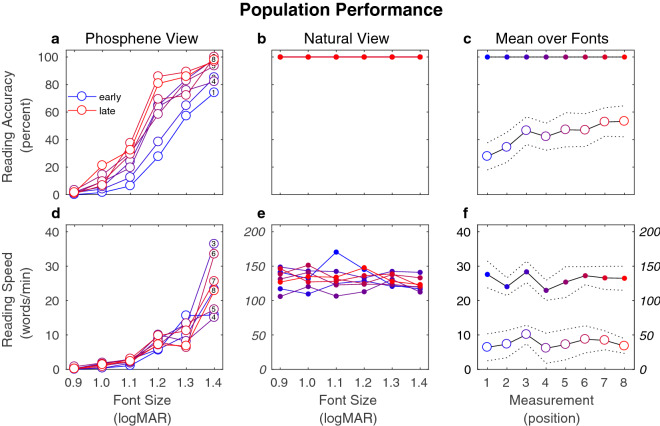
Population performance on reading accuracy and speed. The reading accuracy [(**a**–**c**), in units of percent correct] and reading speed [(**d**–**f**) in units of words per minute] are shown for population means at each of the eight possible measurement positions (colored traces, numbered) in the sequence of sessions. As not all subjects are administered a test at each measurement position in the sequence in order to create the RvvvR/RvvvvvvR spans examined elsewhere, the number of subjects varies from curve to curve here (see Fig. 9). Colors shift from earlier (blue) to later (red) measurements, with each trace in the left column (**a**,**d**) also identified by measurement position. The left (**a**,**d**) and middle (**b**,**e**) columns show curves that are the mean over subjects, while the right column (**c**,**f**) takes an additional mean over font sizes with dashed lines showing standard deviations across subjects. Phosphene view measurements (open circles) show leftward progression of the reading accuracy curves, but no similar progression in reading speed. Natural view measurements that serve as a control condition (closed circles) show normal values for reading accuracy—all overplotted at 100%—and speed without significant learning effects. Note that the lower right subfigure (**f**) has two different scales for the two different curves, left for phosphene view (open circles), and right for natural view (closed circles).

Before exploring the details of results from the two different spans, we examined overall performance across the full sequence of sessions, here for reading accuracy, and in the next section for reading speed. We found that as a population, subjects improved (Fig. 1) in reading accuracy (percentage of words read correctly) through the sessions but did not improve in reading speed (number of correctly read words per minute). As expected, reading accuracy was near zero at the smallest font sizes and followed a sigmoidal increase toward the larger font sizes. Over the suite of eight reading tests the sigmoidal profile shifted leftward toward smaller fonts as ability increased for the population (Fig. 1a). The mean accuracy pooled across font sizes (Fig. 1c) rose significantly from 28 ± 7% to 53 ± 9% (Wilcoxon rank sum, *p* = 0.003) echoing the first part of the learning curve from our previous report^1^.

### Reading speed

Reading speed was also near zero for the smallest font sizes and increased with larger font sizes (Fig. 1d), but did not show evidence of plateauing for the font sizes used here (larger sizes would likely have been necessary), consistent with our previous study. Over the course of the experiment, the curves did not move appreciably for the population. The mean speed pooled across font sizes (Fig. 1f) showed initial hints of improvement over the first three measurements, but did not change significantly following these first measurements (Wilcoxon rank sum, *p* = 0.72, 6.4 ± 3.1 WPM at start, 6.8 ± 1.8 WPM at end).

### Acuity over time

**Figure 2:**
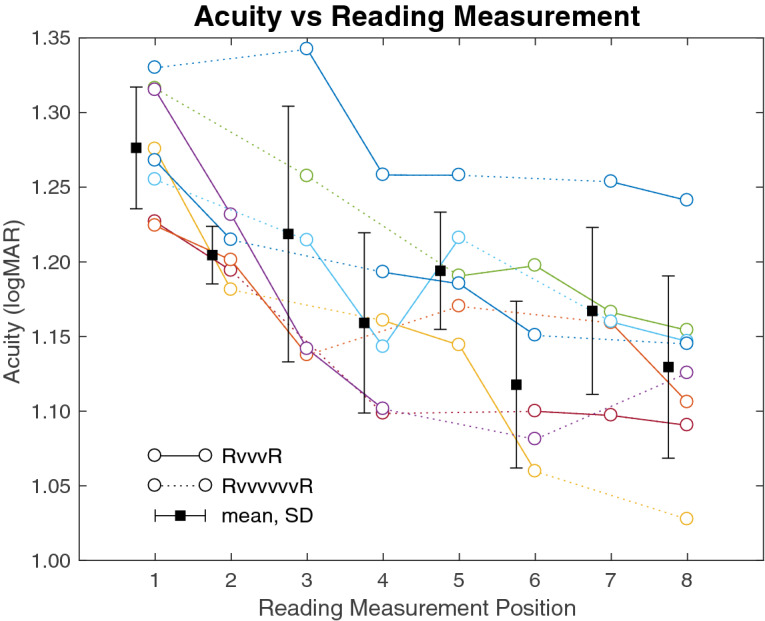
Acuity over time. The equivalent acuity is shown for each reading test for each subject. Each colored trace represents one subject. Acuity measurements from reading tests that are separated by three video sessions (RvvvR) are connected with a solid line, while those separated by six video sessions (RvvvvvvR) are connected with a dashed line. Each subject displays a progression to improved acuity (lower values on vertical axis) through their participation in the experiment (horizontal axis), with the population as a whole moving strongly downward.

Equivalent acuity was then extracted from logistic curves fitted to the reading accuracy measurements from each subject and examined for longitudinal effects (Fig. 2). For each subject, there was a striking change over the eight measurements in a manner equivalent to a statistically significant acuity improvement of − 0.15 ± 0.05 logMAR (*t* test of the paired differences, *p* = 0.0001) from a starting value of 1.28 ± 0.04 logMAR to a finishing value of 1.13 ± 0.06 logMAR. The level of variability in acuity assessments was elevated as expected due to having limited the number of repeated measurements in each reading task in order to minimize the amount of time subjects spent in that activity. Despite the resulting uncertainty in acuity values, there remained a clear effect of improvement in acuity for each subject through the duration of the experiment.

### Relative contributions of R and v sessions to acuity

**Figure 3:**
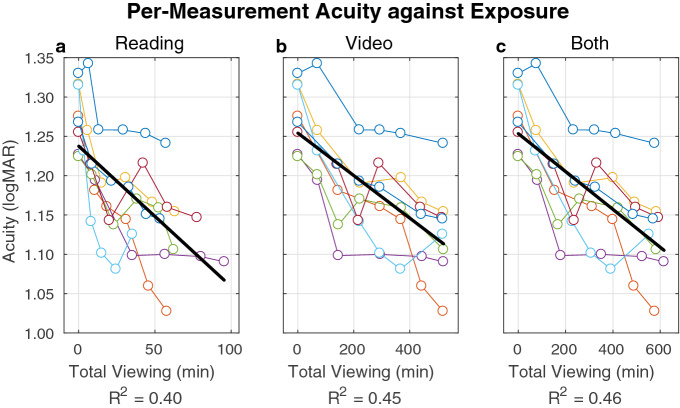
Acuity vs phosphene exposure. Acuity (vertical axes) is shown at each of the six measurements made for each subject (colored lines) is plotted against either the total time spent in the reading task [(**a**), left plot], watching videos [(**b**), middle], or the combination [(**c**), right]. For each plot, a linear regression of the data is shown (heavy black line) and the *R*^2^ coefficient given below the axis. The best fit is delivered when acuity is considered against total viewing time (c, reading and video), with nearly as good a fit with video time alone (**b**), and a somewhat worse fit with reading time alone (**a**), suggesting that learning was driven by both reading and video experiences.

**Figure 4:**
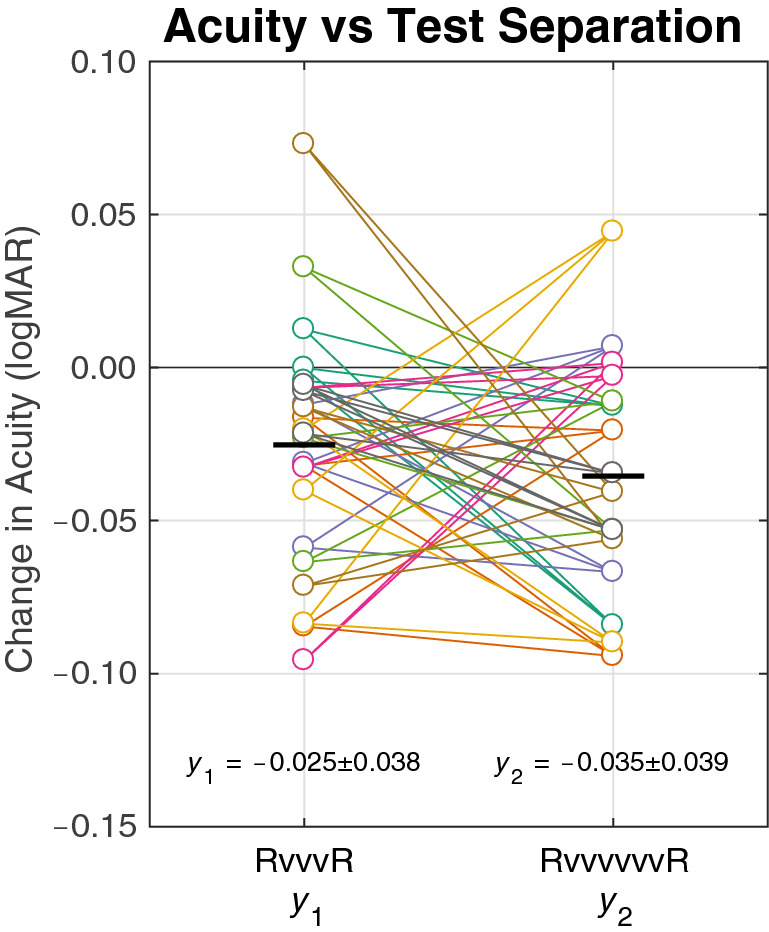
Determining *β*_R_ and *β*_V_ from *y*_*1*_ and *y*_*2*_. Change in reading acuity is shown (open circles), segregated for reading tests separated by three video viewing sessions (RvvvR) and six video viewing sessions (RvvvvvvR) in the syncopated schedule. Pooled measurements for improvements *y*_1_ and *y*_2_ refer to the change in reading acuity between three video viewing sessions (RvvvR) and six video viewing sessions (RvvvvvvR), respectively. Improvements *y*_1_ and *y*_2_ are assumed to contain the influence from one reading session test session *R* (*β*_R_) and the three or six video sessions *v* (*β*_V_) in each span [see Eqs. (1) and (2)]. Links are drawn (colored lines) between pairs of *y*_1_ and *y*_2_ values from the same subject. The mean change in acuity (black bars) for both *y*_1_ and *y*_2_ is negative, indicating an improvement in reading ability, but as both RvvvR and RvvvvvvR spans have the same number of *R* sessions, the larger number of encompassed *v* sessions represented by the value *y*_*2*_ creates a larger improvement as compared to *y*_1_. That difference allows us to deduce the influence of each session type independently, calculating values for *β*_V_ and *β*_R_ through linear decomposition in Eqs. (3) and (4) (see “Decoding acuity gain factors for reading and video sessions”).

We proceeded to analyze the two factors of reading time and video time in driving acuity improvement. An initial assessment showed both factors were predictive of visual acuity (*R*^2^ = 0.39 for reading, *R*^2^ = 0.44 for video, *R*^2^ = 0.45 for reading and video together; *p* < 10^−6^ for all three conditions; see Fig. 3). We then applied a synchronous decoding technique (see “Methods”), sorting the incremental improvements in acuity into short (RvvvR) and long (RvvvvvvR) spans between measurements (Fig. 2) to collect the associated factors *y*_1_ (− 0.025 ± 0.038; *t* test for mean being non-zero, *p* = 0.004) and *y*_2_ (− 0.035 ± 0.039, *p* = 0.002) allowed us to decode the per-session learning rate for the active reading task *β*_R_ = − 0.015 ± 0.084 logMAR/session, and the passive video task *β*_V_ = − 0.003 ± 0.018 logMAR/session (Fig. 4). These values were both significantly non-zero (*t* test for *β*_R_, *p* = 0.001; for *β*_V_, *p* = 0.001) and were statistically distinct (paired *t* test, *p* = 0.02). To validate this method, we also performed a linear regression of acuity versus cumulative time spent reading and viewing video at each acuity measurement, which yielded learning rates of − 0.0006 logMAR/min (+ 0.0005, − 0.0017, 95% CI) for reading and − 0.0002 logMAR/min (+ 0.0000, − 0.0004, 95% CI) for video viewing. When multiplied by the mean session lengths (*t*_reading_ = 13.6 min, *t*_video_ = 24.7 min), this second set of values became *β*_V_′ = − 0.009 logMAR/session and *β*_V_′ = − 0.005 logMAR/session respectively, agreeing reasonably well with our primary finding for *β*_R_ and *β*_V_. We therefore draw two conclusions. First, a single reading session drove about twice the acuity improvement of a single video session despite being about half as long. And second, considering the total number of sessions spent in each task, the overall acuity gain was 56% from reading (mean improvement, − 0.08 logMAR) and 44% from video watching (mean improvement, − 0.06 logMAR).

To ensure the initial transient that can be seen in Fig. 2 was not unduly influencing our results, we repeated the primary synchronous decoding analysis, excluding the first through fourth measurements. While the uncertainty increased and statistical power was lost as there were fewer data points, the results were highly consistent with *β*_R_ being substantially larger than *β*_V_.

### Video viewing behavior

Subjects were not provided guidance on what to do when watching the videos, and some were truly passive, hardly moving their gaze location from the center of the screen, whereas others appeared to be more intently following the on-screen action. We quantified this range of behavior by measuring the total scan path length of gaze location per session and found a trending increase from 279 ± 76 to 338 ± 66 screen widths per video (Wilcoxon rank sum, *p* = 0.1) over the 21 video sessions. A similar, although more significant, increase was found for the reading task with the scan path increasing from 8.4 ± 2.5 to 14 ± 5 screen widths per trial (Wilcoxon rank sum, *p* = 0.003) over the reading sessions (see “Discussion”). The correlation coefficient between the scan path length at each reading session and the subsequent video session, once scan paths were corrected for the per-subject mean, was *r*(46) = 0.42, *p* = 0.02, suggesting the two conditions have a link to an underlying common factor such as session-by-session motivation levels.

### Experimental design validation and control condition

The experiment was intentionally designed to minimize the fraction of time during the reading task, so as to limit the known learning effects from that task against the intended measurement of learning effects during the video task. We were largely successful with the design, as the subjects spent 13 ± 3% of total exposure to the phosphene view simulation in reading (4483 ± 1223 s), as compared to video viewing (30,389 ± 1050 s). The ratio of the mean exposures was therefore 1:6.8, or 1 min of phosphene reading for about every 7 min of video viewing.

Reading in the Natural View condition, with text shown normally in unmodified form on the screen, was our primary control (Fig. 1, see also “Methods”). Reading accuracy at all font sizes was 100% for all subjects in this condition, as expected. Mean reading speed pooled across fonts for the population was not significantly different (Wilcoxon rank sum, *p* = 0.51) between the first (138 ± 15 WPM) and last (132 ± 14 WPM) measurements, and qualitatively did not appear to be affected through the experiment, matching behavior observed in our previous report with a different cohort of subjects drawn from a similar pool^1^.

## Discussion

We found that passive viewing of videos through our simulation of artificial vision was not nearly as effective for improving visual acuity as reading for the same amount of time. Although significant increases in population reading accuracy were observed, significant improvement for reading speed was not apparent. Our initial hypothesis that passive viewing would be a useful tool in rehabilitation was found to be partially correct: while there was a specific increase found in reading skill, namely reading accuracy, it was not reflected in a universal improvement in proficiency with phosphene vision, as an equivalent increase was not found in reading speed. The most compelling interpretation for this dichotomy is a combination of two related aspects, first that our assumption that passive experience would result in an overall sharpening of ability was incorrect, and second that the sets of skills exercised by the reading task only partially overlapped with those exercised by the video task.

Previous work in the laboratory utilizing multiple phosphene patterns has established that effects of training transfer from one pattern to another in an active, letter recognition task^26^. Thus, we might reasonably expect transferability of skill to be present here as well. Despite this expectation, we did not find evidence of transfer from the video viewing task to all aspects of reading skill.

### Comparison of active task here to previous work

The cumulative time spent reading here is 74.7 ± 20.4 min (*n* = 8 subjects), equivalent to the cumulative time in the first four sessions out of the 40 total in our previous experiment^1^ at 77.6 ± 12.6 min (*n* = 6 subjects). Here, there was a significant improvement of − 0.08 ± 0.02 logMAR attributable to the reading task, versus the slightly higher − 0.10 ± 0.08 logMAR for the equivalent time in the previous study. While the two sets of improvements were not significantly different (Wilcoxon rank sum, *p* = 0.49), the possibly higher underlying rate in the previous study may stem from the reinforcing effects of frequent training that were not as strong here due to intentional gaps between reading sessions to reduce that task’s influence.

### Dilution of reinforcement

The larger gaps between reading sessions here, and the weaker influence of video sessions on learning may have diluted reinforcement effects and impeded reading speed development. Reinforcement effects that varied with time between sessions were seen in our earlier work^1^, where reading accuracy was found to be more robust to gaps in training than reading speed. Whereas short gaps of a few days tended to not affect improvements made in reading accuracy, they worked against or eliminated improvements in reading speed such that only a strong gain in a day’s session overcame a low-level of persistent forgetting. With that observation in mind, one explanation for the lack of improvement in reading speed here is that the gain during video viewing, as indicated by the per-session *β*_V_, might not have been large enough to sustain improvements across sessions. Another explanation, perhaps not mutually exclusive, is that the act of reading combines two aspects of visual perception, one of raw acuity used to recognize visual patterns and extract probabilistic information on underlying object shape, and a second of word recognition from that probabilistic shape description; if we assume that the first is trained both under reading as well as video experience, but the second aspect is trained only during reading, then it would be reasonable to see a disparity in reading accuracy versus reading speed as observed here.

### Scan path lengths

Increases in scan path length in the reading task as performance improves are slightly counter-intuitive. We explored this observation through a re-analysis of the more extensive data from our previous report^1^, and found that when subjects are doing very poorly, they generally have short scan lengths in the reading test because they quickly abandon sentences under difficult reading conditions. As their skill improves, they abandon less and spend more time searching and exploring, increasing the scan length, which peaks when the mean performance is 50%. As performance continues to improve, the scan length decreases again as the amount of searching drops and the gaze behavior more closely resembles the left-to-right, line-by-line pattern from normal reading. Thus, we believe the general increase in scan path length seen here reflects a still-continuing improvement in skill like the first part of the more fully completed examples in our previous research. Scan path lengths would therefore be expected to decline again here with additional training. We might imagine that the similar, but less pronounced scan path change observed in the video task would follow a similar up-and-down profile; without prior evidence to support this speculation, however, certainty can come only with additional investigation.

### Transferability of visual skill

In the design of our experiment, we have assumed that there would be a transferability for skills learned during the passive task (specifically, the ability to recognize objects that are viewed through the phosphene pattern), to the active task where our quantitative measurements are made, and thus any specific improvement driven by video viewing would be reflected in a generalized improvement in all aspects of the reading assessment. This idea of transferability, or generalization from one set of experiences to another, has been the subject of substantial investigation in the visual system, often under the umbrella of VPL.

Transfer of visual skills has been observed in such diverse conditions as with subjects impaired by amblyopia^44–48^, central vision loss^49,50^**,** presbyopia^51,52^, low vision in children^53^, macular degeneration^54^, cortical blindness from stroke^55,56^, visual function loss^57,58^, and impaired vision^59,60^. And transfer of perceptual learning has been demonstrated in situations such as in shaping a preferred retinal locus in the visual periphery^50^, moving regions of sensitivity in areas of the visual cortex^54^, improving letter recognition^46,61,62^, seeing biological motion in noise^63^, and eye-hand coordination in sports^64,65^. Such changes are seen especially when training is based on a broad stimulus set^65–67^, retinal locations^56^ or uses video games^67–73^. Training to respond to these stimuli has been reported to improve visual acuity and contrast sensitivity for both central and peripheral vision of subjects^67^, actual field performance of baseball players^65^, as well as reading^73^. The wide palette of modalities where skill transfer has been found suggests that transfer between the two tasks used in this study should be possible.

Observations have been made, however, about limitations or specificity of transfer. For example, McGovern and colleagues observed improvements that transferred between the related visual tasks of orientation, curvature, and global form discrimination after 10 sessions of 400 trials^74^. In particular, both orientation and global form discrimination transferred to the other two tasks, however, the curvature task transferred only to the orientation task. This asymmetry of action demonstrates that visual skill development can be highly specific.

### Transferability in studies with simulations of artificial vision

Narrowing the range of studies to those that are closest to the present report, two groups have studied the transferability of trained skills in simulations of artificial vision. The first group, Sommerhalder and colleagues used a reading task to investigate the generalization of monocularly presented stimuli to stimuli presented to the fellow eye^28,29^. They simulated an eccentric (non-foveal) placement of a visual prosthesis and had normally sighted subjects perform reading tasks with that simulation. Subjects had a series of 1-h sessions reading 4-letter words for a period of 1 month and subsequently 1-h sessions of reading full-page text for a period of 2 months. VPL was observed to be successfully transferred from the trained to the untrained eye.

The second group, Wang and colleagues investigated the transfer between two related tasks, object-to-name labeling and name-to-object identification in a cross-validated experiment using two subject cohorts^75^. In their labeling task, subjects were presented a single object through a simulation of artificial vision and had to choose the correct label out of a set of possible words shown in the clear. In the identification task, subjects were presented instead a single label in the clear and had to choose the correct object out of a set of possible objects, shown in a simulation of artificial vision. In a 1-day session of 64 trials, visual recognition significantly transferred from the labeling task to the identification task with substantial effect in the first cohort but not vice versa in the reverse cohort. In a follow-up study, the group investigated persistence of learning with their labeling task as well as the effect of viewing images compared to videos^76^. Training with the labeling task led to an improved performance, with the improvement with videos being more pronounced than with still images. Next, using their identification task, they found that performance with videos exceeded that with still images in all blocks, but not with as robust a difference. These asymmetries between the nature of both the task and the stimuli confirm the potential for specificity in visual skill development.

### Stutter-step design and recovery of gain factors for R and v

We selected a stutter-step design using two different length runs of video viewing sessions bounded by reading sessions in order to be able to dissect out the relative influences of the reading and video viewing sessions. An alternative design would have been to, for example, employ separate control and experimental groups both of whom would be administered regularly spaced reading assessments, but only the experimental group would have seen videos in between reading assessments (see our previous report describing an experiment with a similar condition^1^, and the comparison here in section “Comparison of active task here to previous work”). A third alternative would be to employ a crossover design where videos would be seen by one half of the subjects in the first half of the schedule, and by the other group in the second half. Neither of these approaches, or of the many others that were considered, would have completely compensated for longitudinal effects while also allowing subjects to be their own controls and, importantly, allowing for the rigorous separation of the two factors *β*_R_ and *β*_V_. The stutter-step strategy selected supports analysis through the method of synchronous decoding developed for signal detection theory, a powerful means to extract signal in the face of noise, allows each subject to be their own control, and maximizes the statistical power available from a limited number of subjects.

### Video watching as a passive task

We selected watching videos as a passive task because we wanted to use an activity that recipients of visual prostheses might experience without the need for a special-purpose rehabilitative apparatus. We reasoned that video watching might be effortlessly and pleasantly incorporated into post-implant daily routines with little prodding required from rehabilitation specialists. While the visual aspect of watching videos was expected to be the primary driver of the effects seen here, we included the audio track of each episode in our simulations to more accurately emulate the experience of a prosthesis recipient sitting in front of a television. We did not, however, explore whether the presence of audio itself was a facilitating factor.

## Conclusions

Although the idea of being able to improve the utility of a visual prosthesis through an enjoyable pastime like watching television programs is highly attractive, in our hands, this process does not have nearly the impact as an active, at times challenging task which provides clear-cut automatic feedback for correct answers. While there appear to be benefits from passively viewing videos, the gains are much slower per unit time, and do not appear to transfer universally to other visual skills. During post-implant rehabilitation, therefore, having patients engage in an active process appears to be preferable to ensure successful treatment with a visual prosthesis device. Further research will help identify self-administered active and passive tasks that overcome specificity to allow broad improvements in prosthetic vision, while being as enjoyable as possible to encourage patient engagement in post-implant rehabilitation.

## Methods

**Figure 5:**
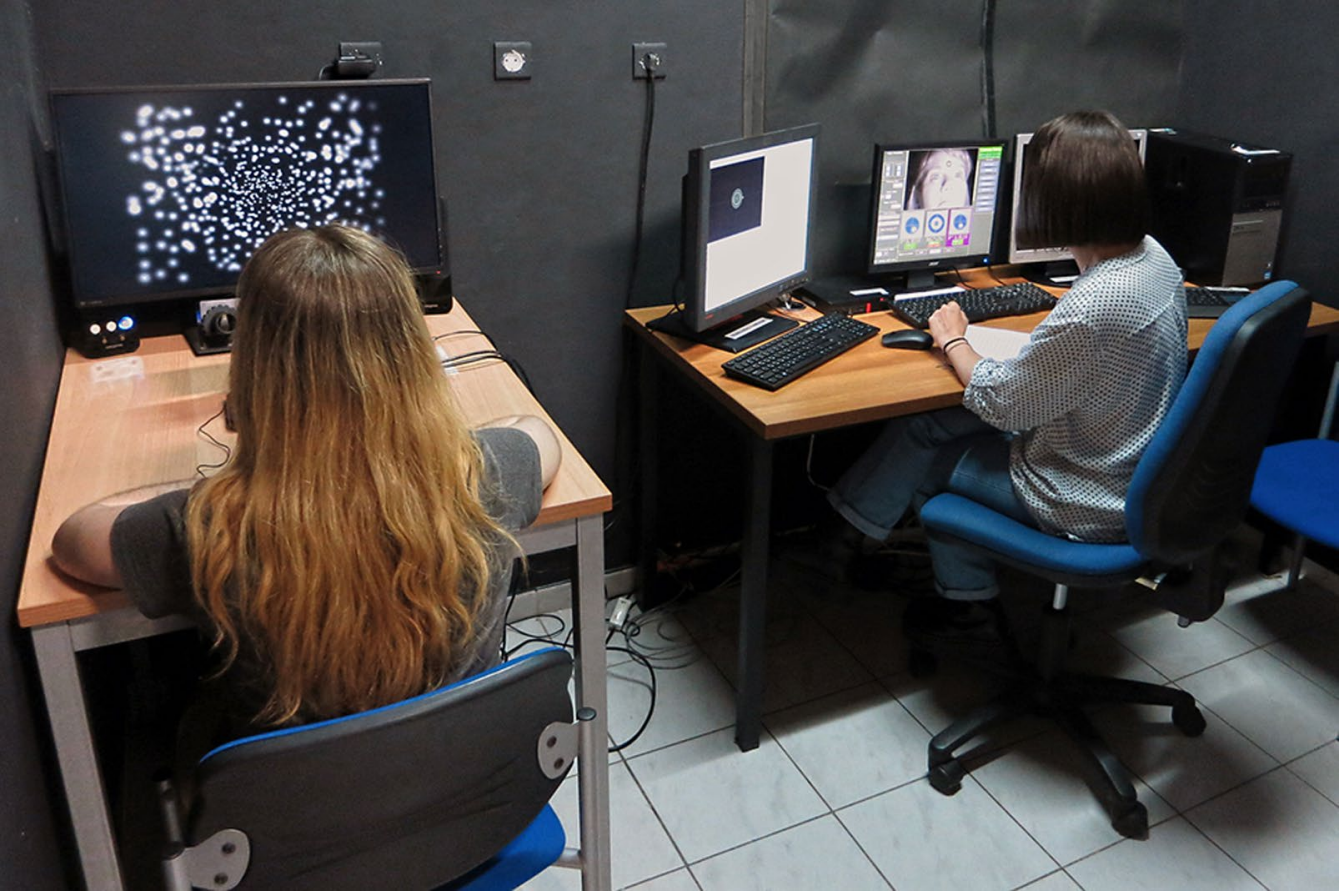
Apparatus. The experimental setup is shown in this photograph with a subject seated on the left in front of the stimulus monitor, and the gaze-tracking camera just visible to the left of their head. The subject’s position with arms on the table helps reduce head movement. The stimulus monitor displays a phosphene-view image of one frame from a video. On the right, the experimenter is sitting in front of the behavioral control system and gaze tracker. This image has been intentionally over-exposed to help reveal detail, as the room is normally dim.

**Figure 6:**
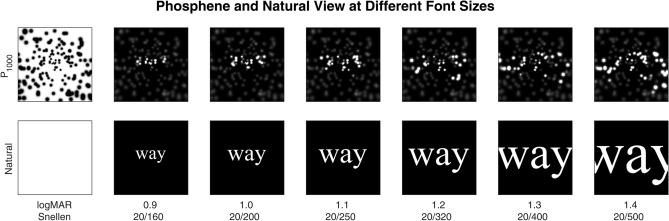
Reading test stimuli. This figure shows the twelve possible stimulus conditions for the example word *way*. The left column depicts the central 10° of viewing although it is presented here in negative colours. Stimuli were always presented to the subjects with white on black. In the right columns, gaze location is at the center of the word so that the highest density of phosphene layout overlays the text. Viewing conditions were either presented in Natural view for control (bottom row) or P_1000_, a simulation of a thalamic visual prosthesis with 1000 phosphenes (top row). Font size ranged from logMAR 0.9 to 1.4, corresponding to a Snellen acuity from 20/160 to 20/500 (left to right). The full three-line simple sentences occupied the entire video screen at the largest font size (see Fig. 7), although only the central-most patch is shown here. The P_1000_ phosphene map spans the entire visual field, although only the central portion is shown here, with some 250 phosphenes.

**Figure 7:**
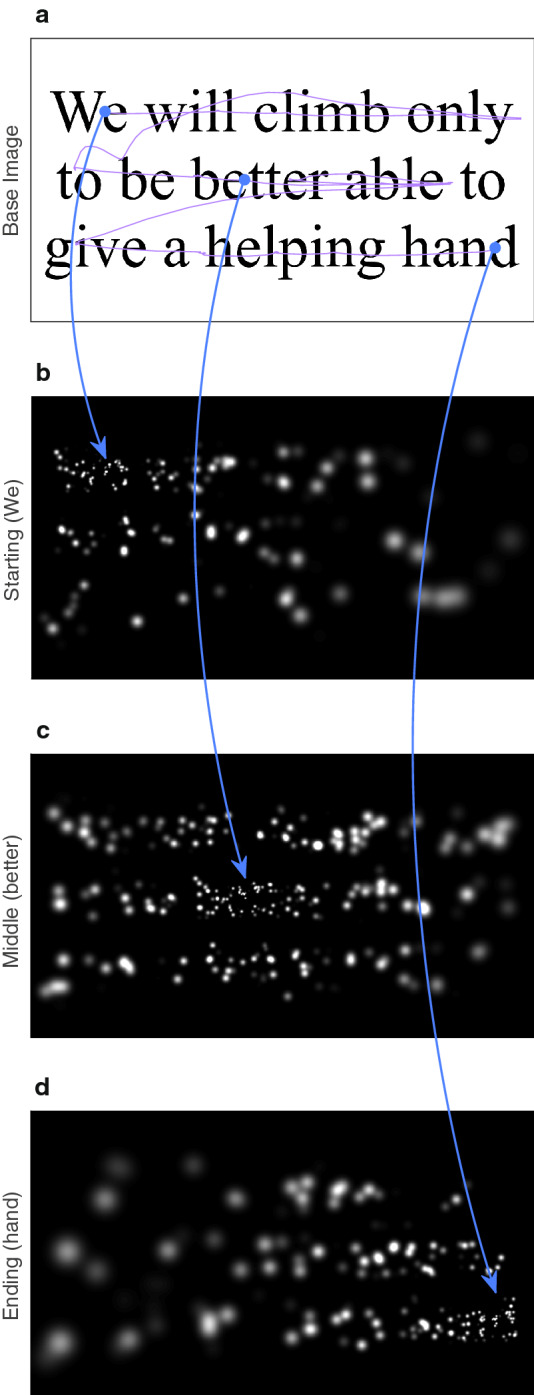
Reading screenshots. These panels depict three points during the reading of a typical sentence, “We will climb only to be better able to give a helping hand,” at the largest font size. The subject reads each of the three lines in turn, scanning along the text. (**a**) Gaze position over time (purple trace) is overlaid on the base image used to compute the phosphene view animation presented to the subject. The base image is never itself visible during phosphene view conditions. Three example points at the start, middle, and end of the scanning are highlighted and connected to their equivalent locations on the lower panels that are snapshots from the trial’s gaze-contingent presentation. (**b**) The phosphene pattern shown to the subject at the start of this trial when the subject is gazing at the start of the first line, and is about to read the word, *We*. (**c**) The subject has scanned through the first line and is mid-way through the second line during this second screen capture. As the subject’s gaze position shifts, the phosphene pattern shifts in rigid concert, and we now see the subject looking at the word, *better*. (**d**) The subject has nearly finished the trial, scanning through all three lines, and is about to read the last word, *hand*. The number of phosphenes landing on the monitor area that are potentially activated are 500, 640, and 430 for subplots, (**b**), (**c**) and (**d)**, respectively. Of these phosphene locations, 260, 400, and 250 have been activated, respectively, to represent the image of the text.

**Figure 8:**
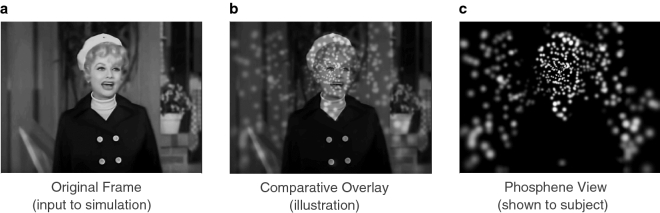
Video stimuli. (**a**) A representative stimulus frame. Video frames are used as input to the simulation along with the current gaze position that determines the location of the phosphene field as it is overlaid on the image. (**b**) An example overlay of the phosphene pattern for the gaze position at the center of the frame. Each phosphene will represent the mean brightness of the image behind it, thus the brightest points are at the woman’s white hat and face, and the darkest are over her coat and portions of the background. The phosphene pattern is updated in position based on gaze location and brightness with each video frame. Phosphenes are smaller and denser toward the point of regard, here near the center of the face, and become larger and more separated in the periphery. (**c**) The resulting constructed phosphene image as shown to the subject. The head and neck of the actress can be made out against larger rectangular fields that correspond to portions of the background. With some effort, the eyes and mouth are discernible—while the example screen shot suggests it might be quite difficult to perceive fine detail, with an animated presentation the experience is surprisingly improved as motion information becomes available to the viewer.

The methods employed in this report are very similar to those previously described^1^ with differences as highlighted below. Briefly, we used a simulation (Fig. 5) that included 1000 phosphenes in a center-weighted pattern^36^ that were activated in a gaze-contingent fashion^77^, tracking instantaneously measured eye position on a frame-by-frame basis so as to approximately stabilize their position in retinal coordinates. The phosphenes were used both as a filter to measure the average image luminance at each phosphene’s location, and then as a display, illuminated as a 2D-Gaussian at the matching luminance. This method is often called *veridical encoding*. The simulation was used to provide two different tasks to the subjects, a reading task that was used as metric of visual performance (Figs. 6, 7), and a video task that was the focus of this work (Fig. 8); the first task requires active participation from the subject, and was designed to be a small fraction of the total experience, while the second task has no specific burden of action from the subject, and was designed to be the dominant portion of the total experience. Each of the two tasks are described below in more detail.

### Differences: extended viewing and visual load

Reading tests here and in our previous work involved brief periods of phosphene viewing (approximately 30 s on average per trial) separated by inter-trial intervals, interleaved with natural viewing and presented through multiple trials per test, whereas video viewing was for a much more extended, continuous time, typically 24 min, that did not include interruptions. The level of subject engagement required was very different between the reading tests and video viewing, as the act of reading demands active focus to understand each word, whereas watching light entertainment has no goal-directed need to parse visual information.

### Differences: more complicated schedule

Although subjects shared the same number of video-viewing sessions, each subject’s six pseudo-periodic measurements of their reading performance took place distributed across eight different possible positions, in a per-subject pattern that was determined beforehand. This pattern resulted in gaps between reading tests of either three (RvvvR) or six (RvvvvvvR) videos, with each subject having three of the shorter gaps and two of the longer gaps.

### Differences: lower system latency, higher monitor refresh rate

In contrast to the previous experiment for which the eye-tracking system was operating at 500 Hz, the monitor’s refresh rate was 60 Hz, and the overall system-latency was expected to be 35 ms total, here, we operated the eye-tracking system (SR Research, EyeLink 1000+) at 1000 Hz, used a monitor that could support 144 Hz refresh rate (Asus ROG PG279Q), optimized the custom simulation code, and estimate the latency from eye position measurement to display update to be 11 ms (1 ms gaze measurement, plus 7 ms frame update, plus 3 ms monitor lag).

### Reading task and stimuli

Reading tests used the MNREAD corpus of simple, three-line sentences presented in a range of font sizes (logMAR 0.9 to 1.4 in 0.1 increments) to assess visual acuity^34,78^, modified so that subjects viewed the sentences shown on a computer monitor either through natural vision as a control condition, or through a simulation of a thalamic visual prosthesis^1,31,35^ with 1000 phosphenes that spanned the visual field^36^. The reading test method has been described previously in detail^1,25,26,31,35^, and is summarized here. In a series of trials, subjects are shown novel sentences from the corpus at different font sizes in a pre-determined, pseudo-random sequence. Each font size and viewing condition is presented twice in a given reading test. The number of correctly read words is scored by the experimenter, and the time taken to read each sentence is recorded automatically. From these values, profiles of reading accuracy (percent of correctly read words) and reading speed (number of correctly read words per minute) are developed across the calibrated font sizes. Reading accuracy versus font size can be fitted with a sigmoidal curve where the 50% level is considered the visual acuity of the subject for the given viewing condition. All font sizes presented were substantially larger than native acuity of the subjects, and thus reading accuracy was 100% for the Natural condition that used text presented without filtering. An example of the range of font sizes against the phosphene pattern is given in Fig. 6, while an example sentence seen under phosphene view with different gaze positions is given in Fig. 7.

### Video stimuli

During video sessions, subjects were presented full episodes of the classic American comedy, “I Love Lucy,” viewed through the visual prosthesis simulation. We used these shows because of the ready availability of the material on recorded media, the light and entertaining subject matter, and the expectation that while our subjects would likely be generally familiar with the series, none of them would have recently, if ever, seen the show. We compiled various publicly available best-of lists to identify the most engaging and popular episodes and selected a suite of 21 that were presented in order. Episodes were shown through our simulation of artificial vision using a real-time gaze-contingent architecture (see below) and were presented with normal audio that was synchronized to the simulation. Videos were typically 24–27 min long, although subjects often broke off their viewing at the closing credits. A representative frame in its original state and as viewed through the simulation is given in Fig. 8.

### Gaze-contingent architecture

Visual stimuli were presented on a contemporary LCD monitor (ROG PG279Q by Asus, Inc.) set to 1600 by 900 pixels resolution and a frame rate of 144 Hz (6.9 ms between frame updates). This monitor was selected because of its high-quality IPS panel, fast refresh rate, and extremely low latency (3.25 ms measured by TFT Central, https://www.tftcentral.co.uk/reviews/asus_rog_swift_pg279q.htm). Video signals sent to the monitor were computed in real-time based on subject gaze position measured through a head-free tracker (EyeLink 1000+, by SR Research, Inc.) running at 1000 Hz. For each monitor refresh, the center of the phosphene pattern was computationally translated to the most recently read gaze location and each phosphene used as a local-averaging filter at its position on the current frame from the television episode to construct a phosphene-view frame displayed on the subject monitor. The ratio between monitor refresh (144 Hz) and episode frame rate (23.96 Hz) of 6.01 meant that each video frame was presented 6 times to the subject, with one frame shown 7 times approximately every 4 seconds to resynchronize; when videos were viewed in the clear during development, these periodic resynchronizations were imperceptible. With this monitor and gaze tracker, we estimate the system latency to have been approximately 10 ms. Additional details of the gaze tracking and filtering process can be found in our previous publications^1,25,26,31,35^.

### Syncopated schedule

**Figure 9:**
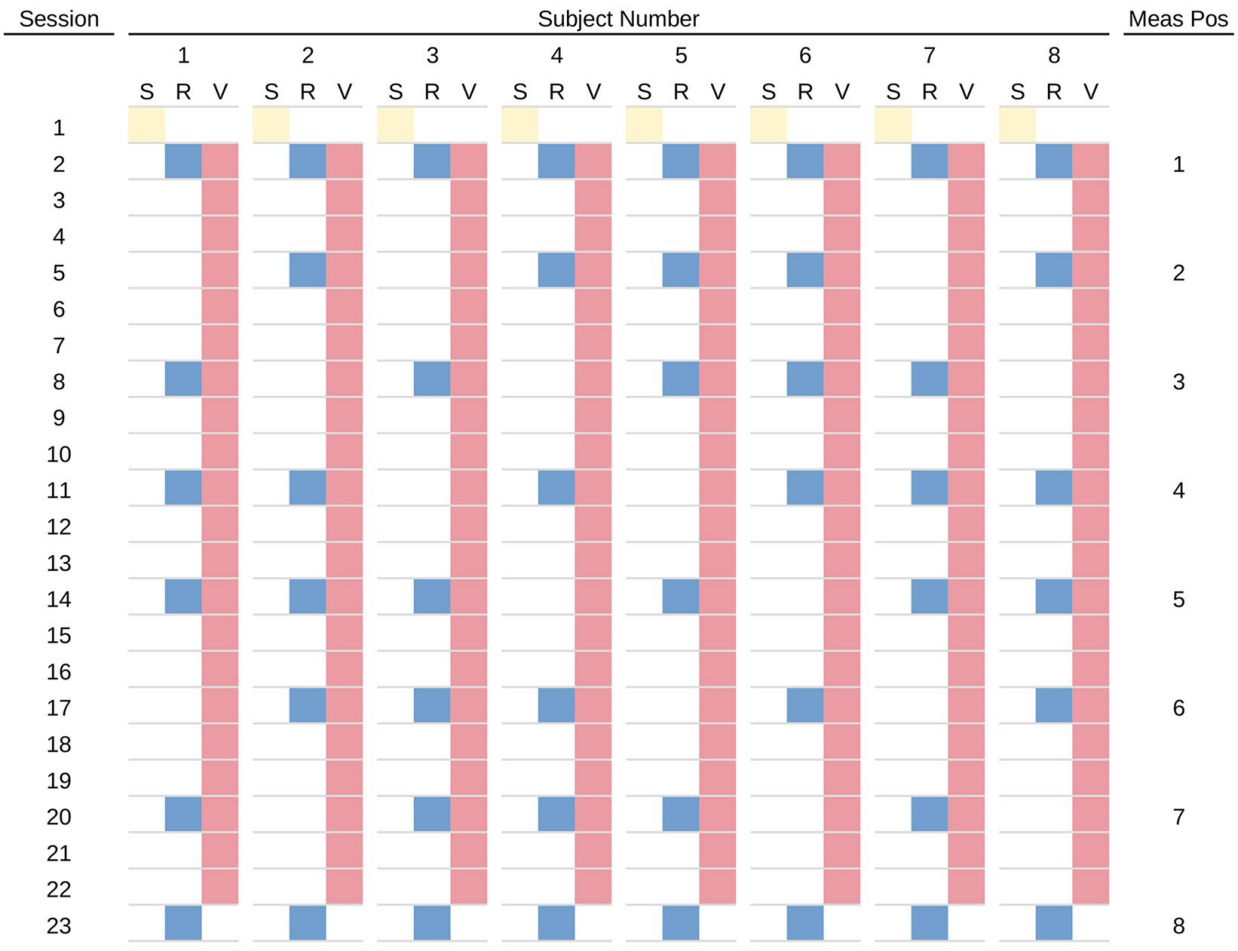
Testing schedule. The testing schedule was used to coordinate the reading tests that were performed every three (RvvvR) or six (RvvvvvvR) video sessions in syncopated fashion across the eight subjects, as shown in this table. Sessions are shown across rows and subjects in groups of three columns, one for each of Snellen acuity test (yellow squares), reading test (blue squares), and video viewing (red squares). Eight potential positions appear for the reading tests starting with Session 2 and ending with Session 23, as shown in the rightmost column. The majority of days for each subject consisted of just video viewing. Each day that there was both a reading test and a video viewing, the reading test was administered first. The three or six video viewings between reading tests were used to drive the decoupling of learning effects from the two tasks.

A pre-determined testing schedule was used, developed on the basis of eight subjects with 23 visits each to the laboratory. At the first session, each subject was given an informal Snellen chart evaluation to verify that they had normal or corrected-to-normal vision. At subsequent sessions they were either given a reading test, or watched an episode of the television series, or both, as determined by the testing schedule. Positions in the schedule were created for reading tests every three sessions of video viewing, with a final test on the last day, alone, for a total of eight potential testing days. We utilized an interleaved syncopation when determining when subjects would be administered reading tests within those eight possible positions such that each subject skipped two tests in the 23 sessions (see Fig. 9). This schedule resulted in gaps between reading tests of either three (RvvvR) or six (RvvvvvvR) video sessions, with each subject having two tests separated by six videos, and three tests separated by three videos. Across the population, the first and last test positions were fully populated and thus produced eight measurements, whereas the second through seventh test positions were partially populated so produced either five or six measurements.

### Decoding acuity gain factors for reading and video sessions

For the short spans between reading tests/acuity measurements, RvvvR, we assumed there would be a constant factor of improvement from *R* (the second *R* measures the influence of the first *R,* but is assumed to carry no influence on its own measurement) and a different, constant factor from each *v*, thus generating an overall improvement *y*_1_:

1 $$y_{{1}} = { 3}\beta_{{\text{V}}} + \beta_{{\text{R}}}$$

For the long spans between measurements, RvvvvvvR, we assumed these same factors would combine with twice as many instances of *v*, to yield an overall improvement *y*_2_:

2 $$y_{{2}} = { 6}\beta_{{\text{V}}} + \beta_{{\text{R}}}$$

These expressions for the measured improvements *y*_1_ and *y*_2_ create a system of two linear equations with two unknowns, allowing us to decode the influence from reading *β*_R_, and video watching *β*_V_:

3 $$\begin{aligned} y_{{1}} -y_{{2}} &= { 3}\beta_{{\text{V}}} + \beta_{{\text{R}}} - \left( {{6}\beta_{{\text{V}}} + \beta_{{\text{R}}} } \right)\\ &= { 3}\beta_{{\text{V}}} + \beta_{{\text{R}}} - { 6}\beta_{{\text{V}}} - \beta_{{\text{R}}} \\ &= - { 3}\beta_{{\text{V}}}\\ \beta_{{\text{V}}} &= \left( {y_{{2}} - y_{{1}} } \right)/{ 3} \end{aligned}$$

4 $$\begin{aligned} {2}y_{{1}} -y_{{{2} }} &= { 2 }\left( {{3}\beta_{{\text{V}}} + \beta_{{\text{R}}} } \right) \, - \, \left( {{6}\beta_{{\text{V}}} + \beta_{{\text{R}}} } \right)\\ & = { 6}\beta_{{\text{V}}} + { 2}\beta_{{\text{R}}} - { 6}\beta_{{\text{V}}} - \beta_{{\text{R}}}\\ & = \beta_{{\text{R}}}\\ \beta_{{\text{R}}} &= { 2}y_{{1}} - y_{{2}} \end{aligned}$$

Equations (3) and (4) were thus used to convert *y*_1_ and *y*_2_ measurements of acuity improvements from one reading test to the next (Fig. 4) into separated values for *β*_V_ and *β*_R_.

We note that accepting this model for extracting *β*_V_ and *β*_R_ from *y*_1_ and *y*_2_ requires understanding that there is no a priori expectation for statistical independence of *y*_1_ and *y*_2_ . For example, in the hypothetical case where video viewing has no influence whatsoever, the value of *β*_V_ would be 0 and the model formed by Eqs. (1) and (2) implies that the observations of *y*_1_ and *y*_2_ would be statistically indistinguishable.

### Ethics statement

The protocol used in this experiment was approved by the Institute Review Board of the Massachusetts General Hospital and the Ethics Board of the Cognitive Sciences Department in the Department of History and Philosophy of Science at the National and Kapodistrian University of Athens. It conformed to the Declaration of Helsinki. The protocol was classified as a minimal risk study with informed consent obtained from each subject. Subjects were provided modest monetary compensation for their participation.

## References

[1] Rassia KEK, Pezaris JS (2018). Improvement in reading performance through training with simulated thalamic visual prostheses. Sci. Rep..

[2] Sasaki Y, Nanez JE, Watanabe T (2010). Advances in visual perceptual learning and plasticity. Nat. Rev. Neurosci..

[3] Skrandies W, Fahle M (1994). Neurophysiological correlates of perceptual learning in the human brain. Brain Topogr..

[4] Clapp WC (2005). Effects of long-term potentiation in the human visual cortex: A functional magnetic resonance imaging study. NeuroReport.

[5] Teyler TJ (2005). Long-term potentiation of human visual evoked responses. Eur. J. Neurosci..

[6] Gutnisky DA, Hansen BJ, Iliescu BF, Dragoi V (2009). Attention alters visual plasticity during exposure-based learning. Curr. Biol..

[7] Beste C, Wascher E, Güntürkün O, Dinse HR (2011). Improvement and impairment of visually guided behavior through LTP- and LTD-like exposure-based visual learning. Curr. Biol..

[8] Clapp WC, Hamm JP, Kirk IJ, Teyler TJ (2012). Translating long-term potentiation from animals to humans: A novel method for noninvasive assessment of cortical plasticity. Biol. Psychiatry.

[9] James KH, Atwood TP (2009). The role of sensorimotor learning in the perception of letter-like forms: Tracking the causes of neural specialization for letters. Cogn. Neuropsychol..

[10] Herzog MH, Fahle M (1997). The role of feedback in learning a Vernier discrimination task. Vis. Res..

[11] Seitz AR, Watanabe T (2003). Is subliminal learning really passive?: Psychophysics. Nature.

[12] Cortese A, Lau H, Kawato M (2020). Unconscious reinforcement learning of hidden brain states supported by confidence. Nat. Commun..

[13] Christou CG, Bülthoff HH (1999). View dependence in scene recognition after active learning. Mem. Cogn..

[14] James KH, Humphrey GK, Vilis T, Corrie B (2002). “Active” and “passive” learning of three-dimensional object structure within an immersive virtual reality environment. Behav. Res. Methods Instrum. Comput..

[15] Meijer F, Van der Lubbe RHJ (2011). Active exploration improves perceptual sensitivity for virtual 3D objects in visual recognition tasks. Vis. Res..

[16] Chrastil ER, Warren WH (2013). Active and passive spatial learning in human navigation: Acquisition of survey knowledge. J. Exp. Psychol. Learn. Mem. Cogn..

[17] Dagnelie G, Walter M, Yang L (2006). Playing checkers: Detection and eye–hand coordination in simulated prosthetic vision. J. Mod. Opt..

[18] Pérez Fornos A, Sommerhalder J, Pittard A, Safran AB, Pelizzone M (2008). Simulation of artificial vision: IV. Visual information required to achieve simple pointing and manipulation tasks. Vis. Res..

[19] Srivastava NR, Troyk PR, Dagnelie G (2009). Detection, eye–hand coordination and virtual mobility performance in simulated vision for a cortical visual prosthesis device. J. Neural Eng..

[20] Josh, H., Yong, B. & Kleeman, L. Mobile, real-time simulator for a cortical visual prosthesis. In *Proceedings of the International Conference on Biomedical Electronics and Devices* 37–46 (SciTePress—Science and Technology Publications, 2012).

[21] Thompson RW, Barnett GD, Humayun MS, Dagnelie G (2003). Facial recognition using simulated prosthetic pixelized vision. Investig. Ophthalmol. Vis. Sci..

[22] Xia P, Hu J, Peng Y (2015). Adaptation to phosphene parameters based on multi-object recognition using simulated prosthetic vision: Phosphene parameters adaptation. Artif. Organs.

[23] Chen SC, Hallum LE, Lovell NH, Suaning GJ (2005). Learning prosthetic vision: A virtual-reality study. IEEE Trans. Neural Syst. Rehabil. Eng..

[24] Fu L, Cai S, Zhang H, Hu G, Zhang X (2006). Psychophysics of reading with a limited number of pixels: Towards the rehabilitation of reading ability with visual prosthesis. Vis. Res..

[25] Bourkiza B, Vurro M, Jeffries A, Pezaris JS (2013). Visual acuity of simulated thalamic visual prostheses in normally sighted humans. PLoS One.

[26] Killian NJ, Vurro M, Keith SB, Kyada MJ, Pezaris JS (2016). Perceptual learning in a non-human primate model of artificial vision. Sci. Rep..

[27] Brindley GS (1964). The number of information channels needed for efficient reading. J. Physiol..

[28] Sommerhalder J (2003). Simulation of artificial vision: I. Eccentric reading of isolated words, and perceptual learning. Vis. Res..

[29] Sommerhalder J (2004). Simulation of artificial vision: II. Eccentric reading of full-page text and the learning of this task. Vis. Res..

[30] Dagnelie G, Barnett D, Humayun MS, Thompson RW (2006). Paragraph text reading using a pixelized prosthetic vision simulator: Parameter dependence and task learning in free-viewing conditions. Investig. Ophthalmol. Vis. Sci..

[31] Vurro M, Crowell AM, Pezaris JS (2014). Simulation of thalamic prosthetic vision: Reading accuracy, speed, and acuity in sighted humans. Front. Hum. Neurosci..

[32] Hayes JS (2003). Visually guided performance of simple tasks using simulated prosthetic vision. Artif. Organs.

[33] van Rheede JJ, Kennard C, Hicks SL (2010). Simulating prosthetic vision: Optimizing the information content of a limited visual display. J. Vis..

[34] Mansfield JS, Legge GE, Luebker AT, Cunningham K (1994). MNREAD Acuity Charts.

[35] Paraskevoudi N, Pezaris JS (2021). Full gaze contingency provides better reading performance than head steering alone in a simulation of prosthetic vision. Sci. Rep..

[36] Pezaris JS, Reid RC (2009). Simulations of electrode placement for a thalamic visual prosthesis. IEEE Trans. Biomed. Eng..

[37] Stingl K (2015). Subretinal visual implant alpha IMS—Clinical trial interim report. Vis. Res..

[38] Zrenner E (2011). Subretinal electronic chips allow blind patients to read letters and combine them to words. Proc. Biol. Sci..

[39] Kapetanovic J (2020). Highest reported visual acuity after electronic retinal implantation. Acta Ophthalmol..

[40] Humayun MS (2009). Preliminary 6 month results from the Argus II epiretinal prosthesis feasibility study. Annu. Int. Conf. IEEE Eng. Med. Biol. Soc..

[41] da Cruz L (2016). Five-year safety and performance results from the Argus II retinal prosthesis system clinical trial. Ophthalmology.

[42] Castaldi E (2016). Visual BOLD response in late blind subjects with Argus II retinal prosthesis. PLoS Biol..

[43] Erickson-Davis C, Korzybska H (2021). What do blind people ‘see’ with retinal prostheses? Observations and qualitative reports of epiretinal implant users. PLoS One.

[44] Levi DM, Polat U (1996). Neural plasticity in adults with amblyopia. Proc. Natl. Acad. Sci..

[45] Levi DM, Polat U, Hu Y-S (1997). Improvement in Vernier acuity in adults with amblyopia. Investig. Ophthalmol..

[46] Polat U (2008). Restoration of underdeveloped cortical functions: Evidence from treatment of adult amblyopia. Restor. Neurol. Neurosci..

[47] Levi DM, Li RW (2009). Perceptual learning as a potential treatment for amblyopia: A mini-review. Vis. Res..

[48] Hussain Z, Webb BS, Astle AT, McGraw PV (2012). Perceptual learning reduces crowding in amblyopia and in the normal periphery. J. Neurosci..

[49] Chung STL (2011). Improving reading speed for people with central vision loss through perceptual learning. Investig. Ophthalmol. Vis. Sci..

[50] Liu R, Kwon M (2016). Integrating oculomotor and perceptual training to induce a pseudofovea: A model system for studying central vision loss. J. Vis..

[51] Polat U (2009). Making perceptual learning practical to improve visual functions. Vis. Res..

[52] Polat U (2012). Training the brain to overcome the effect of aging on the human eye. Sci. Rep..

[53] Nyquist JB, Lappin JS, Zhang R, Tadin D (2016). Perceptual training yields rapid improvements in visually impaired youth. Sci. Rep..

[54] Baker CI (2005). Reorganization of visual processing in macular degeneration. J. Neurosci..

[55] Huxlin KR (2009). Perceptual relearning of complex visual motion after V1 damage in humans. J. Neurosci..

[56] Das A, Tadin D, Huxlin KR (2014). Beyond blindsight: Properties of visual relearning in cortically blind fields. J. Neurosci..

[57] Fronius M, Cirina L, Cordey A, Ohrloff C (2005). Visual improvement during psychophysical training in an adult amblyopic eye following visual loss in the contralateral eye. Graefe’s Arch. Clin. Exp. Ophthalmol..

[58] Ostrovsky Y, Andalman A, Sinha P (2006). Vision following extended congenital blindness. Psychol. Sci..

[59] Huang C-B, Zhou Y, Lu Z-L (2008). Broad bandwidth of perceptual learning in the visual system of adults with anisometropic amblyopia. Proc. Natl. Acad. Sci..

[60] Zhou J (2012). The eye limits the brain’s learning potential. Sci. Rep..

[61] Chung STL, Legge GE, Cheung S (2004). Letter-recognition and reading speed in peripheral vision benefit from perceptual learning. Vis. Res..

[62] Polat U, Ma-Naim T, Belkin M, Sagi D (2004). Improving vision in adult amblyopia by perceptual learning. Proc. Natl. Acad. Sci..

[63] Grossman ED, Blake R, Kim C-Y (2004). Learning to see biological motion: Brain activity parallels behavior. J. Cogn. Neurosci..

[64] Clark JF, Ellis JK, Bench J, Khoury J, Graman P (2012). High-performance vision training improves batting statistics for university of Cincinnati baseball players. PLoS One.

[65] Deveau J, Ozer DJ, Seitz AR (2014). Improved vision and on-field performance in baseball through perceptual learning. Curr. Biol..

[66] Xiao L-Q (2008). Complete transfer of perceptual learning across retinal locations enabled by double training. Curr. Biol..

[67] Deveau J (2014). Broad-based visual benefits from training with an integrated perceptual-learning video game. Vis. Res..

[68] Green CS, Bavelier D (2003). Action video game modifies visual selective attention. Nature.

[69] Green CS, Bavelier D (2007). Action-video-game experience alters the spatial resolution of vision. Psychol. Sci..

[70] Li R, Polat U, Makous W, Bavelier D (2009). Enhancing the contrast sensitivity function through action video game training. Nat. Neurosci..

[71] Green CS, Li R, Bavelier D (2010). Perceptual learning during action video game playing. Top. Cogn. Sci..

[72] Li RW, Ngo C, Nguyen J, Levi DM (2011). Video-game play induces plasticity in the visual system of adults with amblyopia. PLoS Biol..

[73] Deveau J, Seitz AR (2014). Applying perceptual learning to achieve practical changes in vision. Front. Psychol..

[74] McGovern DP, Webb BS, Peirce JW (2012). Transfer of perceptual learning between different visual tasks. J. Vis..

[75] Wang L, Sharifian F, Napp J, Nath C, Pollmann S (2018). Cross-task perceptual learning of object recognition in simulated retinal implant perception. J. Vis..

[76] Wang L, Marek N, Steffen J, Pollmann S (2021). Perceptual learning of object recognition in simulated retinal implant perception—The effect of video training. Transl. Vis. Sci. Technol..

[77] Paraskevoudi N, Pezaris JS (2019). Eye movement compensation and spatial updating in visual prosthetics: Mechanisms, limitations and future directions. Front. Syst. Neurosci..

[78] Mansfield JS, Atilgan N, Lewis AM, Legge GE (2019). Extending the MNREAD sentence corpus: Computer-generated sentences for measuring visual performance in reading. Vis. Res..

